# Current Trends of Bioactive Peptides—New Sources and Therapeutic Effect

**DOI:** 10.3390/foods9070846

**Published:** 2020-06-29

**Authors:** Anna Jakubczyk, Monika Karaś, Kamila Rybczyńska-Tkaczyk, Ewelina Zielińska, Damian Zieliński

**Affiliations:** 1 Department of Biochemistry and Food Chemistry, University of Life Sciences in Lublin, 20-704 Lublin, Poland; monika.karas@up.lublin.pl; 2 Department of Environmental Microbiology, University of Life Sciences in Lublin, 20-069 Lublin, Poland; kamila.rybczynska-tkaczyk@up.lublin.pl; 3 Department of Analysis and Evaluation of Food Quality, University of Life Sciences in Lublin, 20-704 Lublin, Poland; ewelina.zielinska@up.lublin.pl; 4 Department of Animal Ethology and Wildlife Management, University of Life Sciences in Lublin, 20-950 Lublin, Poland; damian.zielinski@up.lublin.pl

**Keywords:** bioactive peptides, antioxidant, metabolic syndrome, peptide inhibitors, antimicrobial peptides, peptides from edible insects

## Abstract

Generally, bioactive peptides are natural compounds of food or part of protein that are inactive in the precursor molecule. However, they may be active after hydrolysis and can be transported to the active site. Biologically active peptides can also be synthesized chemically and characterized. Peptides have many properties, including antihypertensive, antioxidant, antimicrobial, anticoagulant, and chelating effects. They are also responsible for the taste of food or for the inhibition of enzymes involved in the development of diseases. The scientific literature has described many peptides with bioactive properties obtained from different sources. Information about the structure, origin, and properties of peptides can also be found in many databases. This review will describe peptides inhibiting the development of current diseases, peptides with antimicrobial properties, and new alternative sources of peptides based on the current knowledge and documentation of their bioactivity. All these issues are part of modern research on peptides and their use in current health or technological problems in food production.

## 1. Introduction

Nowadays, food is considered as a source of not only dietary compounds but also biologically active compounds that may exert a beneficial effect on human health and condition of the organism. The growing consumers’ awareness of the impact of diet on health is reflected in their choice of raw products rich in vitamins, minerals, and other bioactive compounds such as polyphenols [1], anthocyanins [2], carotenoids [3,4], essential oils [5], or peptides [6].

**Figure 1:**
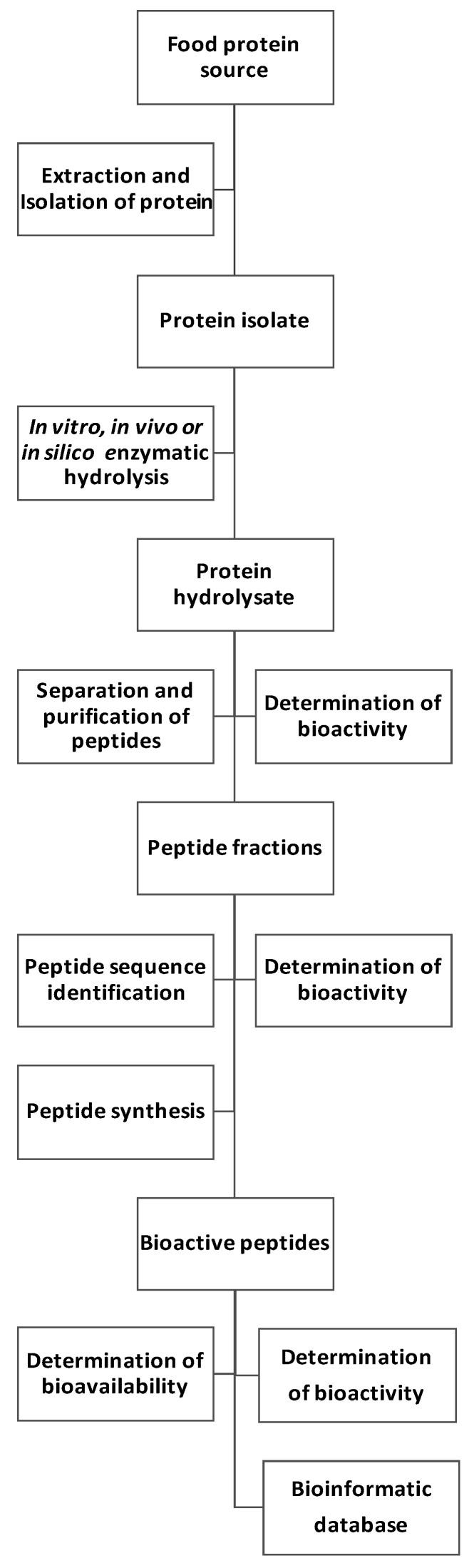
Scheme of bioactive peptide preparation.

Biologically active peptides may be natural compounds of food or part of protein that are inactive in the precursor molecule but are active after release or are transported to the active site [7]. Generally, peptides are a result of enzymatic hydrolysis of protein in the gastrointestinal tract. They can also be produced by microorganisms in the fermentation process. To obtain biopeptides with specific activity, proteases with broad specificity of action are used for proteolysis. They are extracted from vegetable tissues (e.g., ficin, papain, bromelain), animal tissues (e.g., pepsin, chymotrypsin, trypsin), and microbial cells (e.g., proteinase K, pronase, collagenase, subtilisin A, Alcalase^®^, Flavourzyme^®^, Neutrase^®^) [8]. There are many methods to obtain peptides with biological properties and increase the isolation process (Figure 1). Several enzymes of different origin are also used to isolate new, most often short-chain, peptides. Before hydrolysis, the source of peptides may be subjected to various processes, e.g., fermentation [9,10,11]. Biopeptides can also be synthesized chemically [12,13,14] or can be obtained through the expression of appropriate genes [15,16,17].

**Figure 2:**
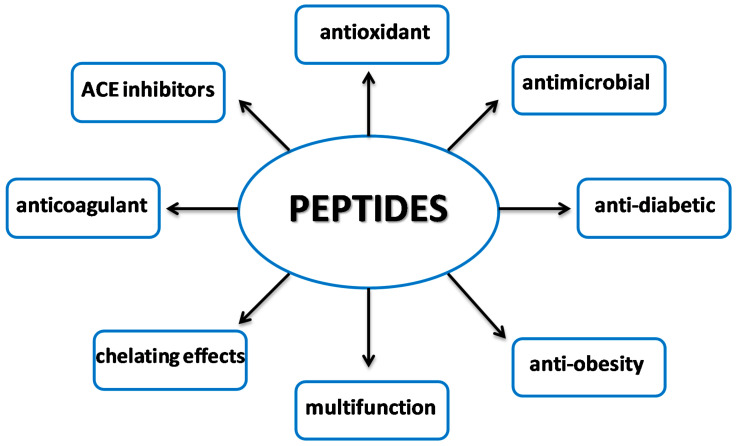
Properties of bioactive peptides.

Peptides have been investigated for a long time and it is currently known that, similar to hormones, they can regulate many important body functions. They are characterized by many properties, including antihypertensive, antioxidant, antimicrobial, anticoagulant, and chelating effects (Figure 2). They are also responsible for the taste of food or inhibition of enzymes involved in the development of diseases. However, peptides may also show toxic activity or allergenic effects, especially in people with celiac disease. The activity of peptides depends on their structure and amino acid composition. Until recently, it was thought that bioactive peptides are composed of 2–20 amino acids. It is now known that they may contain more amino acids in their structure (for instance, insulin with the peptide-C structure), which play a special role in carbohydrate metabolism; hence, analysis of their content is an important factor in diabetic diagnosis [8].

Protein hydrolysates and peptide fractions with determined molecular mass or peptides can be used as functional foods, nutraceuticals, or additives to food products, increasing their nutraceutical potential. Good sources of peptides are protein-rich food products of plant [18], animal [19], or alternative origin [20,21]. Recently, peptides were isolated and identified from products with low content of protein [22], new foods [23], residues from food production [24], or non-food products [25]. It seems crucial to find ways for making the best use of such sources of peptides, for example, by using efficient methods to obtain these compounds. An important aspect limiting the use of bioactive peptides is their characteristic bitter taste, which is why they are not always accepted by consumers.

## 2. Peptides as Inhibitors of Enzymes Involved in Metabolic Syndrome

### 2.1. Metabolic Syndrome

One of most common health problems in developing countries is metabolic syndrome (MS). It is estimated to affect approximately 24% of the US adult population. Approximately 47 million people have metabolic syndrome: 44% of those in the ≥50-year age group, 12–37% of the Asian population, and 12–26% of the European population suffer from this disease [26], and many others meet the criteria but have not been correctly diagnosed and are not aware of the disease. There are a few guidelines and diagnostic criteria, but MS is generally defined as a cluster of interconnected metabolic abnormalities involving glucose metabolism, lipid metabolism, cardiovascular blood pressure, and central obesity [27]. MS increases the risk of hypertension, arteriosclerosis-induced heart attack, hypercholesterolemia, dyslipidemia, and type-2 diabetes. It is also related to other disorders, including prothrombotic and proinflammatory processes. It should be noted that MS is increasingly being suggested as a cause of cancer. The main cause of the occurrence and development of the disease is incorrect metabolism associated with excessive activity of certain enzymes.

### 2.2. ACE Inhibitory Peptides

The renin–angiotensin–aldosterone system (RAAS) is a key element in the organism’s homeostasis, control of fluid and electrolyte balance, and response to physiological and pathological conditions. It can act locally at different tissue levels, or the components of the system can be transported by the blood stream into the active site [28]. The most important enzyme in this system is the angiotensin-converting enzyme I (ACE) produced by lung or kidney tissue and the luminal membrane of vascular endothelial cells and other organs. ACE converts inactive decapeptide angiotensin I (ANG I) into vasoconstrictor octapeptide angiotensin II (ANG II). Excessive activity of ACE causes production of high concentrations of angiotensin II and, consequently, an increase in blood pressure. ANG II contributes to multiple physiological and pathophysiological cardiovascular functions such as hypertension, atherosclerosis, thoracic aortic aneurysms, or abdominal aortic aneurysms [29]. Inhibitors of ACE activity are commonly used as drugs in hypertension treatment, but they may cause serious side effects, e.g., cough, rush, or edema [29]. Therefore, identification of new, nontoxic, safe, and inexpensive ACE inhibitors is the main aim of many studies. ACE inhibitors have been isolated and described from protein-rich food products and natural bioresources.

Generally, ACE inhibitory activity is strongly dependent on the amount and type of amino acid composition of peptides, but the exact relationship between their structure and activity is still poorly understood. There are many studies describing ACE inhibitory peptides as short chain molecules, often composed from 2 to 3 amino acids. A novel ACE-inhibitory peptide (VQY) was obtained and identified from razor clams. This peptide with an IC50 of 9.8 µM is one of the competitive ACE inhibitors [30]. A dipeptide (YV) with ACE inhibitory properties was obtained from ostrich egg white ovalbumin. It exhibited an IC50 value of 63.97 μg/mL and was classified as a competitive inhibitor as well. Moreover, the molecular docking analysis revealed that the binding between YV and the S1 and S2 pocket sites of ACE was mainly stabilized by a hydrogen bond [31]. Short chain ACE inhibitory peptides with sequences KVF, MKR, AKF, AMK, and GIL were isolated from enzymatic hydrolysates of lysozyme [32]. Several studies have indicated that binding to ACE is influenced by hydrophobic amino acid residues (aromatic or branched chain) at three positions from the C-terminus of the peptide [33]. Such amino acids as isoleucine and valine in the aliphatic amino acid chain have been reported to enhance the inhibitory effect [34]. Lee and Hur [35] described the amino acid composition as a possible cause of the antihypertensive activity of LIVGIRCV. Hydrophobic amino acid residues with aliphatic side chains, such as G, A, V, L, and I, at the C-terminus have been associated with a significant increase in the ACE inhibitory activity due to the higher binding ability with ACE than in the case of other amino acids. Peptides LPRL, YADLVE, LRLESF, HLNVVHEN, and PGSGCAGTDL obtained from mung bean protein with strong ACE inhibitor activity contained leucine in their structure. The results indicate that this amino acid within the peptide sequence has a main effect on ACE inhibitory activity [36]. In turn, Zhang et al. [37] described two antihypertensive peptides containing tryptophan at the C-terminus—SAGGYIW and APATPSFW, with IC50 values of 0.002 and 0.036 mg/mL, respectively. These results show that the ACE inhibitory property may be associated with tryptophan at the C-terminus, blocking the active site in the enzyme via electrostatic, hydrophobic, van der Waals, and hydrogen bond interactions.

It should be noted that the positively charged amino acids (L and R) have also been involved in the stronger potency of ACE inhibitory peptides. Moreover, hydrophilic amino acids in peptides induce weak or no ACE inhibition [38]. However, in the literature, there are reports on ACE inhibitory peptides CRQNTLGHNTQTSIAQ from *Stichopus horrens* [39] and YGKPVAVPAR, investigated by synthesizing four structural analogs including YHR-10 (YGKHVAVHAR), GA-8 (GKPVAVPA), GHA-8 (GKHVAVHA), and PAR-3 (PAR). It was reported that GA-8 (GKPVAVPA) [12] or NMAINPSKENLCSTFCK obtained from casein [40] did not have any specific amino acids at the C-terminal. This demonstrates that the exact relationship between the amino acid sequence in peptides and their activity is not exactly known.

In addition to the amino acid composition, molecular mass determines the activity of peptides. Research results indicated that peptides with molecular mass above 3 kDa exhibited higher ACE inhibitory activity in comparison with larger peptides [41].

Besides, peptides with more than 2 or 3 amino acids have been identified as ACE inhibitors. Wang et al. [31] described peptides with sequences GHIITVAR, IGGIGTVPVGR, HIGNILSL, FMPGVPGPIQR, PNYHPSPR, AFPAGAAHW, HIITLGR, LAGNPAGR, MPGVPGPIQR, AGALGDSVTVTR, and INTLSGR obtained from sesame protein. These peptides were characterized by molecular weight ranging from 754.8 to 1198.4 kDa, and their IC50 value was in the range 3.60 to 149.63 µM.

Peptides must be unchanged to be delivered to the active site if they are to be used in the treatment of hypertension or heart disease. They should be resistant to digestion by gastrointestinal enzymes or must be transported in capsules or pills to the target site and released there. There are several models reflecting the conditions in the body that are a useful alternative to in vivo conditions. They allow determination of the influence of diet compounds on enzyme activity, digestibility, bioavailability, release of bioactive compounds, and structural changes in food [42]. The model used depends on the food matrix and the aim of study [43,44,45].

There are several studies of peptide ACE inhibitors and their antihypertensive effect in spontaneously hypertensive rats. A potent ACE inhibitory peptide obtained from marine *C. ellipsoidea*, with an amino acid sequence VEGY (MW: 467.2 Da, IC50 value: 128.4 µM), was characterized as a competitive inhibitor against ACE. The peptide was stable against gastrointestinal enzymes. The analysis of the antihypertensive effect in spontaneously hypertensive rats (SHR) also revealed that oral administration of the purified peptide can decrease systolic blood pressure significantly [46]. Lee and Hur [35] reported that a peptide fraction obtained from beef myofibrillar proteins with molecular mass <3.0 kDa (400 and 800 mg/kg body weight treatment groups) induced a decrease in systolic blood pressure by 28 and 35 mmHg, respectively, in a spontaneously hypertensive rat model. The purified peptide ACE inhibitor had a sequence LIVGIIRC. In turn, a study of ACE inhibitors obtained from whey protein reported three potent ACE-inhibitory peptide sequences (DKVGINYW, DAQSAPLRVY, and KGYGGVSLPEW). The highest inhibitory activity was exhibited by KGYGGVSLPEW. A further study indicated no effect of this peptide (5 mg/kg body weight) on systolic, diastolic, and mean blood pressure in spontaneously hypertensive rats (SHR) after single oral administration. Moreover, this peptide had a similar effect to that of ANG II, i.e., it facilitated noradrenaline release from sympathetic nerve terminals. The absence of an antihypertensive effect may also be a result of the interaction of these compounds with other components of the systems involved in the blood pressure control [47]. These results show that peptides with ACE inhibitory activity should also be tested in vivo, as identification and characterization of new peptides are the first steps in determination of their potential use in the treatment of hypertension and cardiovascular disease.

### 2.3. Pancreatic Lipase Inhibitory Peptides

Obesity and fatty acid metabolism disorders are a global epidemic in adults and increasingly in children. They are generally a result of long-term imbalance between energy intake and expenditure and, consequently, the main factor of an increased risk of a cardiovascular disease and type 2 diabetes. It is estimated that the proportion of the population with overweight and obesity in 2030 will reach 89% and 85% in males and females, respectively [48]. There are several mechanisms explaining the relationship between elevated blood pressure and obesity (and caloric excess). One of them is enhancement of the real absorption of sodium or expansion of the intravascular volume, activation of the RAA system by e.g., release of angiotensinogen from adipose tissue, and insulin resistance [49].

Inhibition of the digestion of dietary lipids is one of the strategies for pharmacological intervention, since it does not involve a central mechanism of action. Pancreatic lipase is the main enzyme hydrolyzing 50–70% of food-derived fat in the human organism. Inhibition of this enzyme is the basis of the action of the Orlistat drug used in obesity treatment. Although it exerts satisfying effects on weight control, it has been found to cause serious side effects, especially in long-term treatment (e.g., pancreatic damage, gastrointestinal toxicity, or high cancer risk) [50]. Therefore, it is necessary to search for new safe compounds, also those derived from food, which will be highly effective in pancreatic lipase inhibition without side effects. The literature provides few studies of peptide lipase pancreatic inhibitors. The activity of pancreatic lipase inhibitors depends on the structure of the peptide and amino acid composition. Peptide amphiphiles, which consist of a hydrophilic head group with a bioactive peptide sequence and a chemically conjugated hydrophobic tail, have been proposed for their potential therapeutic applications [51]. In turn, a study conducted by Siow et al. has indicated that the combination of both hydrophilic and hydrophobic properties allows the peptide to act as an inhibitor. The authors suggest that a peptide with a “hydrophilic head” (consisting of RH) and a hydrophobic tail (consisting of PAQPNYPWTAVLVF), will act in the same way. Stefanucci et al. [52] described two peptides with a sequence IWS and YFS exhibiting potent inhibitory activity. Results of another study have demonstrated a peptide with an amino acid sequence CQPHPGQTC, which effectively inhibits pancreatic lipase and is, thus, a promising starting point for development of a new drug [53]. Similarly, two synthetic peptides purified from soybean with sequences EITPEKNPQLR and RKQEEDEDEEQQRE have been described as pancreatic lipase inhibitors (IC50 = 79.27 and 16 μM, respectively) [54]. Our previous results indicated that peptides with sequences GQLGEHGGAGMG, GEHGGAGMGGGQFQPV, EQGFLPGPEESGR, RLARAGLAQ, YGNPVGGVGH, and GNPVGGVGHGTTGT were characterized by pancreatic lipase inhibitory activity. The most effective were GQLGEHGGAGMG and GEHGGAGMGGGQFQPV, with IC50 values 60.62 and 62.32 µg/mL, respectively. It should be noted that the N- and C-termini contain hydrophobic amino acids that may be involved in this activity [14].

As can be seen from these examples, the exact relationship between the inhibitory activity and peptide structure has not been fully elucidated and further studies are needed [55].

### 2.4. Peptide Inhibition of Diabetes Risk Factors

One of the metabolic disorders is type 2 diabetes mellitus. It is estimated that from 221 million to 366 million people worldwide will have been affected by the disease by 2030 [56]. This metabolic disorder is caused by an imbalance in glucose absorption and insulin secretion, leading to higher than normal blood glucose levels [57]. One of the therapeutic methods to reduce blood glucose is to limit its release from food products and absorption. Glucose is released from polysaccharides digested by the α-amylase and α-glucosidase enzymes, which leads to a rapid increase in its blood levels known as postprandial hyperglycemia [58]. Besides stimulation of endogenous insulin secretion, one of the methods to increase the hyperglycemic effect is dietary starch degradation by glucosidases [59]. The common antidiabetic drugs used for diabetes treatment inhibit the release of glucose from food polysaccharides. However, some compounds contained in these drugs, such as acarbose, may cause serious side effects, especially often gastrointestinal symptoms e.g., abdominal distension, flatulence, and diarrhea [60].

Peptides can strongly inhibit α-amylase and α-glucosidase. Peptides with amino acid sequences CSSV (MW = 393.99 Da), YSFR (MW = 570.99 Da), SAAP (MW = 343.89 Da), PGGP (MW = 325.99 Da), and LGGGN (MW = 415.99 Da) were found to possess α-amylase inhibitory activity with IC50 values of 13.76 × 10^3^, 10.82 × 10^3^, 4.46 × 10^3^, 4.23 × 10^3^, and 2.86 × 10^3^ μg/mL, respectively. The IC50 values for α-glucosidase were 206.00, 162.00, 66.90, 63.50, and 42.93 μg/mL, respectively. It should be noted that the LGGGN peptide showed higher inhibition of both α-amylase and α-glucosidase and can, thus, be considered as a potential antidiabetic inhibitor [55]. Wang et al. [61] demonstrated that LPLLR inhibited both α-glucosidase and α-amylase, and the inhibition rates reached a maximum of 50.12% and 39.08%, respectively, at a concentration of 2000 μM. Peptides with α-amylase inhibitory activity were identified in basil seeds [62]. These were P1 (ACGNLPRMC), P2 (ACNLPRMC), and P3 (AGCGCEAMFAGA). According to the in silico structural model, these peptides bound to the substrate binding residues (Trp58, Trp59, Tyr62, Val163, His299, Asp300, and His305) and the catalytic residue (Asp300) of α-amylase with their active fragments (i.e., Asn-Leu-Pro-Arg-Met-Cys of P1 and P2, and Met-Phe-Ala-Gly-Ala of P3). Another example of α-glucosidase inhibitory peptides is those obtained from soy protein, with sequences LLPLPVLK, SWLRL, and WLRL and with IC50 of 237.43 ± 0.52, 182.05 ± 0.74, and 162.29 ± 0.74 μmol/L, respectively [63]. Furthermore, an α-glucosidase inhibitor was purified from *Aspergillus oryzae* N159-1. Analysis indicated that the inhibitor was a tripeptide PFP with the molecular weight of 360.1 Da. The IC50 value of the peptide against α-glucosidase activity was 3.1 mg/mL, and the compound represented a mixed-type inhibitor [25].

The studies of the design of novel α-amylase and α-glucosidase inhibitors are based on their hydrogen bonding interactions and binding energy, which is comparable with that of acarbose. It has been proposed that the structure of such peptides should contain tri- to hexapeptides with serine, threonine, tyrosine, lysine, or arginine as the ultimate N-terminal residue, proline preferably at the penultimate C-terminal position, and alanine or methionine at the ultimate C-terminal position. There are no specific requirements related to peptide hydrophobicity and charge [64]. Other examples of peptides are shown in Table 1.

## 3. Antioxidant Peptides

Many scientific studies indicate that oxidative stress can be the cause of many civilization diseases (obesity, diabetes, heart disease, and cancer). Oxidative stress is caused by an imbalance between production and removal of oxygen reactive species (ROS) in cells and tissues. ROS are generated in normal aerobic cellular metabolism and can play several physiological roles (cell signaling) [76]. However, many environmental stressors (pollutants, heavy metals, and UV or ionizing radiations), xenobiotics, excessive caloric intake, high-fat diets, or progression of aging can contribute to a substantial increase in ROS products, and thus, cause imbalance that leads to cell and tissue damage. Moreover, oxidative stress plays an important role in aging and various neurological disorders in the human organism (such as Alzheimer’s and Parkinson’s diseases) [77]. Therefore, antioxidant compounds play an important role in prevention of free radical-induced tissue damage. In recent years, food proteins have been investigated as a source of peptides with multibiological functions, which promote health and prevent oxidative stress [78,79]. A growing number of antioxidant peptides (Table 2) have been identified from animal, plant, and insect sources, as well as food-processing by-products [21,80,81]. The potential application of food protein-derived antioxidant peptides as additives, nutraceuticals, and pharmacological agents depends on their absorption. Since there are many physiological and biochemical barriers to the absorption of peptides, many solutions have been tested to take advantage of their positive properties fully [7]. From a biological point of view, antioxidant peptides can be divided into endogenous and exogenous. Endogenous peptides occur naturally in cells (glutathione, carnosine, anserine, GHL), while exogenous peptides are derived e.g., from food proteins during gastrointestinal digestion [80,82]. Bioactive peptides can also be released from food proteins during hydrolysis by different proteases of plant, animal, and microbial origin in the food-processing or fermentation process [79,81,83].

### 3.1. Structure-Activity Relationship in Peptides

In general, peptides with low molecular weight and with hydrophobic and aromatic amino acids in their structure have better antioxidant activity; yet, the relationship between the antioxidant activities and structural characteristics of peptides is still not clarified in detail. Antioxidant peptides are oligopeptides including 2–20 amino acids in their structure [82]. The types of amino acids in the peptide sequence have a major impact on their activity. For instance, hydrophobic amino acids with nonpolar aliphatic groups (Y, L, W, P, I, and V) were found to effectively scavenge radicals in foods with high lipid content [84,85,86]. Aromatic amino acid residues (W, Y, and F) in peptide sequences may affect the ability to chelate pro-oxidant metal ions [84,87]. Among them, W, Y, and M had higher antioxidant activity than other amino acids [88]. The presence of hydrophobic (I and L), acidic (D), and basic (H) amino acids in peptide sequences (LDDPVFIH and VAAGRTDAGVH) is believed to contribute to the high antioxidant activity of fermented anchovy fish (Budu) extracts [88]. Moreover, the presence of His in peptide sequences supports their strong antioxidant properties [86]. Lu et al. [87] used a comparative molecular field analysis (CoMFA) model to show a positive correlation between C, M, the bulky C-terminal amino acid residue (R), the negatively charged group around sulfur-containing amino acids, and the antioxidant activity of a nanopeptide with the SYPTECRMR sequence obtained from sesame protein. The authors indicate that sulfur-containing amino acids (C, M) as well as steric and electrostatic factors determine the antioxidant activity of SYPTECRMR. The presence of C, R, E, Q, G, L, and A residues in the structure of RCLQ and EVGK peptides obtained from duck plasma proteins may explain their high antioxidant activities [89]. Wu et al. reported that the high antioxidant potency of QMDDQ from a shrimp protein hydrolysate might be attributed to the greater number of active hydrogen sites and functional groups. Carboxyl active hydrogen atoms and amino active hydrogen atoms as active sites play a critical role in the antioxidant capacity [90].

The composition of the N- and C-terminal regions can be another key factor for the antioxidant ability of peptides. García-Mora et al. [91] demonstrated that the electronic, hydrophobic, and steric properties of amino acid residues in the C-terminal region were important structural features of LLSGTQNQPSFLSGF, NSLTLPILRYL, and TLEPNSVFLPVLLH, determining their high antioxidant properties. Luo et al. [91] showed that an increase in dietary valine levels in young grass carp was accompanied by an increase in the reduced glutathione (GSH) content and the activities of Cu/Zn superoxide dismutase (SOD1) and catalase (CAT). Moreover, Zhang et al. [92] reported that VEVYLPR and VVEVYLPR peptides obtained from egg-white protein exhibited lower antioxidant activity than VYLPR, which explains the fact that the position of glutamate (E) in VEVYLPR and VVEVYLPR may affect their antioxidant activity. Zhang et al. [92] concluded that valine at the N-terminus of VYLPR might be helpful to enhance the antioxidant activity of this peptide. Yang et al. [89] showed that the crucial antiradical activity against ABTS^+•^ of a novel peptide ATVY was dependent on Tyr positions in the N-terminus. In turn, Sheng et al. [93] reported that the presence of antioxidant amino acids (W, Y, and M) did not determine the cellular antioxidative effect of peptides. On the other hand, Matsui et al. [94] concluded that the antioxidant activities of tyrosine containing peptides GYG, GYYG, and YGGY varied, depending on the characteristics of ROS and/or RNS. The author showed that the number and position of Y in the peptides did not affect the antioxidant activity against the ClO^−^ ion, whereas the Tyr position was an important factor for the activity against ONO_2_^−^. In the case of ROO^•^, the number of Tyr residues influenced the antioxidant activity, while its position did not have a significant effect.

The amino acid sequence of peptides determines their bioactivity potential, but it depends on the method used to test antioxidant properties. This was confirmed in a study reported by Sheng et al. [82]. Peptides obtained from walnut meal hydrolysates had similar in vitro antioxidative effects, but differed in the capacity for hydroxyl radical scavenging or ROS reduction. Peptides LAGNPHQQQQN and HNLDTQTESDV had a similar hydroxyl radical scavenging capacity but exerted significantly different in vitro antioxidative effects [82]. In a study conducted by Liang et al. [84], eleven *M. oleifera* seed peptides containing eight dipeptides and three tripeptides showed strong antiradical activities towards DPPH^•^ and ABTS^+•^. Moreover, dipeptides SF and QY showed the best antioxidant activity, which reconfirmed the finding that short peptides have better antioxidant activities. Hydrophobic amino acids play a major role in scavenging free radicals, whereas polar amino acids are responsible for reducing and chelating metal ions [85]. Seven novel peptides—LDGP, TGVGTK, EVGK, RCLQ, LHDVK, KLGA, and AGGVPAG—with antioxidant activities were obtained from duck plasma proteins, identified, and synthesized by Yang et al. [89]. EVGK exhibited the highest Fe^2+^ chelating ability, whereas RCLQ had the highest reducing power and ABTS^+•^ and DPPH^•^ scavenging activity. Four novel peptides with high antioxidant activity were identified by Zhang et al. [82], among which, IREADIDGDGQVN, PEILPDGDHD, and ASDEQDSVRL showed the highest DPPH^•^ scavenging capacity, while APLEEPSSPH had the highest Fe^2+^ chelating ability. Additionally, all novel peptides identified by Zhang et al. [82] had several hydrophobic and/or net-charged residue side chains exposed to the external medium, which was beneficial for their antioxidative capacity. Two antioxidant peptides (TSSSLNMAVRGGLTR and STTVGLGISMRSASVR) were identified by Agrawal et al. [95] from a finger millet protein hydrolysate. Molecular docking studies revealed that the potential antioxidant activity of both peptides resulted from the interaction of Ser and Thr residues with free radicals. Li et al. [96] characterized the primary sequence as well as the secondary and tertiary structures of duck breast protein, deriving peptides using Nano-LC-ESI-LTQ-Orbitrap MS/MS, NICOLET IS10 FT-IR, and the PEPstr server, respectively. AGPSIVH was the best DPPH^•^ scavenger, whereas FLLPH and LLCVAV were effective ABTS^+•^ scavengers. Additionally, LLCVAV proved to have reducing power. Li et al. [96] used FT-IR spectroscopy to estimate the secondary structures of eleven synthesized peptides and suggested that the spatial structure could play a significant role in the antioxidant ability.

### 3.2. Stability and Bioavailability of Antioxidant Peptides

Such structural properties of peptides as the amino acid composition, peptide size, or hydrophobicity determine not only their reactivity, but also their stability and bioavailability [81]. Peptide–food matrix interactions should also be considered, as they can lead to chemical modifications affecting the bioaccessibility and bioavailability of bioactive peptides [7,97]. In this regard, simulated in vitro gastrointestinal (GI) digestion systems are commonly applied for investigation of the release of potentially bioactive peptides from food proteins and for determination of their bioaccessibility [81,82] and the stability of specific antioxidant peptides against simulated GI digestion [81,82,96,98]. Gallego et al. [81] evaluated the effect of in vitro GI digestion on the antioxidant activity of peptides naturally generated in Spanish dry-cured hams within 12 months of processing. Their results showed that the antioxidant activity in the digested samples decreased when measured with DPPH^•^ scavenging activity and ferric-reducing antioxidant power methods, but increased in the ABTS^+•^ scavenging assay. Three novel potent antioxidant peptides AGPSIVH, FLLPH, and LLCVAV were obtained from duck breast protein hydrolysates by Li et al. [96]. The peptides had no toxic effects but exhibited digestive resistance. Interesting information about the intestinal absorption, bioavailability, and pharmacokinetics of antioxidant peptides was reported by Xu et al. [99]. The authors used a model of Caco-2 cell monolayers and oxidative stress in HUVECs to study the absorption and potential antioxidant activity of WDHHAPQLR derived from rapeseed protein. Additionally, an animal model was used to study the metabolism of WDHHAPQLR in vivo and to validate the Caco-2 cell model. WDHHAPQLR was hydrolyzed by intracellular Caco-2 cell enzymes to DHHAPQLR, WDHHAP, and QLR; moreover, these peptides were also detected in rat serum after oral administration. Xu et al. [99] concluded that WDHHAPQLR, DHHAPQLR, and WDHHAP were transported by the paracellular pathway and QLR was transported by PepT1. The absolute bioavailability of WDHHAPQLR was estimated at 3.56%.

### 3.3. Investigations of Cellular Antioxidant Activity

The antioxidant properties of peptides are most often expressed as free radical scavenging (DPPH^•^, ABTS^+•^, or OH^•^) and metal ion chelation (Fe^2+^ and Cu^2+^) activity, inhibition of lipid peroxidation, reducing power, and their influence on the activity of enzymes involved in the oxidation–reduction process (e.g., SOD, CAT, PPO, and GSH-Px) [100]. Recently, the activity of antioxidant peptides has been studied in different cell lines such as HepG2 (hepatocarcinoma), Caco 2 (intestinal cells), HUVeCS (human umbilical vein endothelial cells), and Chang liver cells [84,101]. Several models e.g., AAPH-, H_2_O_2_-treated HepG2, H_2_O_2_-treated Caco 2, and H_2_O_2_-treated SH-SY5Y/HUVeCS cells have been used to investigate the cellular antioxidant activity of peptides [93,100]. Eight peptides with the highest antioxidant activity were selected from hazelnut meal hydrolysates by Shang et al. [93]. In the next stage of their study, the in vitro antioxidant effects of peptides were evaluated in H_2_O_2_-injured SH-SY5Y cells. The results showed that all peptides exerted a protective effect on the proliferation of H_2_O_2_-injured SH-SY5Y cells. Moreover, seven peptides significantly decreased the amount of intracellular ROS. New peptides identified from fermented milk by Tonolo et al. [101] were synthesized and analyzed in vitro and in a cellular model to determine their antioxidant properties. Four of these novel peptides (NTVPAKSCQAQPTTM, EDELQDKIHPF, QGPIVLNPWDQVKR, and APSFSDIPNPIGSENSE) exerted antioxidant effects on Caco-2 cells both via protection against oxidative stress induced by TbOOH and inhibition of ROS production. Novel antioxidant peptides VYLPR, EVYLPR, VEVYLPR, and VVEVYLPR were also identified [92] from egg-white protein. The results obtained by Zhang et al. [92] showed that the peptide VYLPR exerted the strongest protective effect on H_2_O_2_-induced cell damage (HEK-293 cells). Liang et al. [84] investigated antioxidant peptides from a protein hydrolysate of *Moringa oleifera* seeds and their protective effects on Chang liver cells exposed to H_2_O_2_ oxidative damage. Eight novel antioxidant peptides GY, PFE, YTR, FG, QY, IN, SF, and SP and three known antioxidant peptides YFE, IY, and LY were obtained, but only SF and QY showed significantly protective effects on H_2_O_2_-induced Chang liver cells. It was observed that SF and QY increased the activities of endogenous antioxidant enzymes such as SOD and CAT and intracellular ROS scavenging capacity. The authors suggested that SF and QY could potentially serve as natural antioxidants in pharmaceutical products or functional foods (Liang et al., 2020). In turn, Jiang et al. [84] identified four peptides AYI(L) and DREI(L) from Jiuzao protein hydrolysates. The antioxidant activities of these peptides were measured using ABTS, DPPH, ORAC, RP, and FCA assays and in the HepG2 cell model. AYI, AYL, and DREI exhibited strong capacities in the oxygen radical absorbance capacity (ORAC) assay. Their antioxidant capacities were reflected in their ability to decrease ROS in HepG2 cells, enhance the activities of SOD, CAT, and GSH-Px in HepG2 cells, and exert protective effects on AAPH-induced changes in the GSH, GSSG, and MDA contents. In addition, each of the peptides with the same concentration exerted different effects on the activities of SOD, CAT, and GSH-Px [84].

### 3.4. Multifunctional Nature of Antioxidant Peptides

Peptides are often multifunctional and may exhibit several activities such as antioxidative, antihypertensive, anti-inflammatory, cytoprotective, and antimicrobial effects [102]. In a study conducted by He et al. [102], three rapeseed protein-derived ACE inhibitory peptides (LY, RALP, and GHS) were used to investigate their antioxidant and anti-inflammatory properties. For this purpose, a RAW 264.7 cell model and a spontaneously hypertensive rat model were employed. The results showed that LY, RALP, and GHS significantly inhibited the secretion of nitric oxide, interleukin-6, and tumor necrosis factor-α in lipopolysaccharide-stimulated RAW 264.7 macrophages in vitro. In vivo, these peptides inhibited the release of nitric oxide and the production of lipid peroxides and ROS. They also improved cell damage caused by oxidative stress in spontaneously hypertensive rats. As suggested by He et al. [102], LY, RALP, and GHS can protect the organism from oxidative and inflammatory damage. Song et al. [98] observed a positive correlation of the antioxidant and antibacterial activities of cottonseed protein hydrolysates with the contents of basic amino acids (Arg and Lys) and a negative correlation with acidic amino acids (Glu and Asp). García-Mora et al. [91] showed that peptides (LLSGTQNQPSFLSGF, NSLTLPILRYL, and TLEPNSVFLPVLLH) from lentil storage proteins (vicilin, convicilin, and legumin) had dual highest antioxidant and ACE inhibitory activities, and the GI digestion of these peptides improved their dual activity. Moreover, based on molecular docking studies, the authors demonstrated that the C-terminal heptapeptide residues of peptides interacted by hydrogen bonds with three ACE residues of the catalytic site (Tyr520, Lys511, and Gln281).

An important aspect of many studies of bioactive peptides is the selection of protein hydrolysis conditions to obtain compounds with the best specific activities.

Hussein et al. [91] optimized hydrolysis parameters such as the temperature, enzyme/substrate (E/S) ratio, pH, and hydrolysis time, which allowed the obtaining of a whey protein concentrate (WPC) hydrolysate with the highest dual activities. The authors investigated the whey protein concentrate as a unique source of peptides with dual functionalities, i.e., ACE inhibitory and antioxidant activities. The results showed that the selected hydrolysis parameters are very important for the production of hydrolysates with desirable levels of activities. Additionally, Hussein et al. [91] designed the response surface methodology (RSM) for establishment of optimal conditions of hydrolysis of WPC by alcalase to produce protein hydrolysates with the highest activity. It was found that the experimental data, which were well fitted to the predicted data, further validated the regression model adequacy. Antioxidant and cytoprotective peptides were obtained by Ballatore et al. [103] in a process of trypsin-assisted hydrolysis of whey protein concentrate. Peptides with molecular weights lower than 3 kDa exhibited high HO^•^ radical scavenging activity and high cytoprotection against oxidative stress generated by MEN in IEC-18 cells. Therefore, the authors claim that the enzymatic hydrolysis of whey protein concentrate (WPC 35) to produce antioxidant peptides is an innovative approach that can provide added value to whey.

### 3.5. Bioinformatics Studies of Antioxidant Peptides

Currently, many scientific reports contain information from not only in vitro or in vivo tests but also in silico analyses. There are many free online bioinformatic tools that have become popular techniques for investigation and identification of potentially bioactive peptides [91]. An interesting tool used for estimation of the antioxidant properties of peptides are databases, e.g., BIOPEP [104]. BIOPEP is a good software tool for prediction of biological activity and flavor [104]. In turn, QSAR (quantitative structure–activity relationships), QSPR (quantitative structure–property relationships), and molecular docking simulation models can be used for the characterization of structural and physicochemical properties [81]. The authors suggested that in vitro and in silico approaches can be operated in parallel and can be complementary, since they both determine the extracted/predicted peptide and its activity in a complex food matrix. Knowledge of SAR is valuable and useful for estimation of the potential antioxidant activity of peptides derived from food proteins [97]. The SAR analysis may also be applicable in the design of a generation of antioxidant peptides from food proteins as a result of the activity of enzymes. Five novel antioxidant peptides with 10–13 amino acid residues released from the Crucian carp were identified by Zhang et al. [82]. In silico assessments of these peptides showed their amphiphilic nature, good sensory quality, and different target sites in the human body. The authors used the PepDraw tool for the calculation and prediction of hydrophobicity and the net charge of the peptides. The ExPASy ProtParam tool allowed estimation of the instability index, aliphatic index, and GRAVY (grand average of hydropathicity), while the BIOPEP database helped to determine the sensory quality and biological activity of the peptides. Based on the in silico analysis, Zhang et al. [82] suggested that the high antioxidant activity of peptides might be associated with their predicted 3D structure exhibiting at least one β-turn, β-sheet, and/or α-helix with partial hydrophobic and/or net-charged residues exposed to the external medium. A study conducted by Selamassakul et al. [79] focused on the in vitro biological properties of rice protein-derived peptides in relation to in silico flavor characteristics. Most of the eight peptides identified showed ACE inhibitory and antioxidant activity, particularly peptides with the FGGSGGPGG and FGGGGAGAGG sequences. The authors evaluated the flavor characteristics of the peptides using the BIOPEP database. The study results demonstrated high frequencies of umami peptides (ESDVVSDL, GSGVGGAK, and SSVGGGSAG) and a low Q-value.

Recently, antioxidant peptides have become attractive again due to their multiple sources and significant antioxidant activity. Antioxidant peptides are often multifunctional and may exhibit other bioactivities, such as antihypertensive, anti-inflammatory, or antimicrobial effects; therefore, they are attractive substances to be used as food ingredients in enhancing human health. In vitro, in silico, and in vivo research should be conducted in parallel to provide a general view of extracted/predicted peptides and their activity in a complex food matrix or organism.

## 4. Peptides with Antimicrobial Properties

Antimicrobial peptides (AMPs) are generally classified as antibacterial (Table 3), antifungal, and antiviral compounds. The physiological mechanism of antibacterial peptides consists of binding to bacterial cell membranes or mitochondrial membranes, which causes their disintegration and, consequently, cell death. This mechanism is based on the electrostatic interaction between the positively charged peptides and the negatively charged surface of the cell membrane. Then, the peptides disrupt the continuity and structure of the cell membrane [105]. The antimicrobial activity of peptides is related to their physicochemical properties as well as the number and type of amino acids [106,107]. The physicochemical properties of peptides, such as their size, charge, hydrophobicity, amphipathicity, and solubility, are crucial for their antimicrobial properties [107,108]. AMPs are usually rich in cationic and hydrophobic amino acids and have cationic (positively charged) and amphiphilic (both hydrophilic and hydrophobic) characteristics [107]. Additionally, one of the key features of antibacterial peptides is their high content of cysteine and/or glycine residues [109]. Due to their ability to produce a secondary structure, lack of Arg residue in the sequence, or low hydrophobicity, short peptides (4–7 amino acid residues) probably have lower antibacterial activity [106].

## 5. New Alternative Sources of Peptides

The mechanism of the antifungal action of AMP is associated with lysis of fungal cells and disintegration of their cell wall, which affects its permeability. The antiviral effect of some AMPs depends on their interaction with the membrane by electrostatic association with the negative charges of glycosaminoglycans facilitating binding of AMP and competing with viruses [105]. One of the most important antibacterial properties of peptides is inhibition of the growth of foodborne pathogens. Foodborne pathogens cause a great number of diseases with serious effects on human health. Most food poisoning reports are associated with bacterial contamination, especially by members of Gram-negative bacteria like *Salmonella typhi*, *Shigella dysenteriae,* and *Escherichia coli* and Gram-positive bacteria e.g., *Staphylococcus aureus, Listeria monocytogenes*, and *Bacillus cereus* [111,113]. A previous study indicated antibacterial activity of peptide 35,409 against *E. coli* ML 35 (43,827), *Pseudomonas aeruginosa* ATCC 15,442 and *S. aureus* ATCC 29,213 with MIC 22 and 350 µM, respectively [112]. Cusimano et al. [114] demonstrated antibacterial activity of synthetic peptides H2 and Tag against different strains of *L. monocytogenes* (MIC > 5 mg/mL). The antifungal activity of peptides against yeast, e.g., *Candida albicans*, is also important. *C. albicans* yeasts are commensal microorganisms commonly residing on the skin, gastrointestinal tract, genitourinary system, oropharynx, and upper respiratory tract, without causing harm to healthy individuals [110,115]. However, in some cases, they are associated with opportunistic infections in humans, especially in immunocompromised patients with HIV/AIDS [110,116]. In susceptible patients, *C. albicans* can enter the bloodstream by translocation across the mucosa of the gastrointestinal tract [115]. The research conducted by Lum et al. [110] indicated that hybrid peptides KU2 and KU3 containing a mixed backbone of KABT-AMP and Uperin 3.6, demonstrated anticandidal activity against *C. albicans* 90,028 with MIC values in the range of 16–128 mg/L (0.016–0.128 mg/mL). Cyclotides are a large family of plant-derived peptides characterized by a broad range of biological roles, e.g., antiviral activities against viruses involved in human diseases, such as human immunodeficiency virus (HIV), influenza H1N1, and dengue (DENV) [117]. Other plant-derived peptides that can inhibit HIV reverse transcriptase activity are phaseococcin and sesquins isolated from runner bean seeds (*Phaseolus coccineus*) and ground beans (*Vigna sesquipedalis*), respectively [118,119].

### 5.1. Peptides from Edible Insects

Recently, bioactive peptides have been extracted through enzymatic hydrolysis of vegetable proteins e.g., from soya, pea, chickpea, or other popular high-protein plant seeds [78,120]. Nevertheless, new sources of peptides are gaining in popularity, and edible insects are one of the most widely studied sources. Insect consumption is becoming increasingly popular in Europe, and scientists supporting the development of entomophagy as well as producers are trying to make this type of food more attractive to consumers. Therefore, recently, there has been a stronger focus on the pro-health aspect of insects than on their nutritional effect [121]. Since they are a good source of protein, insects can be expected to be a good source of bioactive peptides as well. The research on insect-derived bioactive peptides is relatively new. Nongonierma and FitzGerald [121] indicated that the first publication describing the generation of bioactive peptides from edible insects was published by the University of Ghent in Belgium in 2005 [122]. Bioactive peptides derived from insect proteins are characterized by a wide range of properties such as antioxidant, antimicrobial, antidiabetic, anti-inflammatory, and ACE-inhibitory activities [123]. Insect species that produce bioactive peptides are representatives of the orders Orthoptera, Coleoptera, Lepidoptera, Blattodea, Isoptera, and Hymenoptera [121].

One of the first and most frequently studied insect peptides were derived from the protein of the species *Bombyx mori*, and ACE inhibitory properties have been mostly identified [121,124,125]. The ACE-inhibitory peptides identified from protein of *B. mori* pupae were e.g., KHV [126], ASL [127], and GNPWM [124] (Table 4). Peptides showing this activity were also identified in cricket (*Gryllodes sigillatus*), mealworm (*Tenebrio molitor*), and locust protein (*Schistocerca gregaria*) [44,128]. Peptides inhibiting the activity of other enzymes, e.g., DPP-IV [128], α-glucosidase [44,129], and lipase [44], were identified in insect protein as well. Generally, insects are a source of bioactive peptides with in vitro inhibitory activity against selected enzymes such as ACE, lipase, and α-glucosidase, which may be involved in the pathogenesis of metabolic syndrome. Moreover, the heat treatment of insects has a significant impact on the extraction of bioactive peptides through enzymatic hydrolysis [44].

Another group is insect bioactive peptides reducing inflammation [130]. These peptides have the ability to inhibit lipoxygenase and cyclooxygenase-2 activity. Dual 5-LOX/COX inhibitors induce an enhanced anti-inflammatory effect and act by blocking the formation of both prostaglandins and leukotrienes without affecting lipoxin formation. In addition, such combined inhibition prevents damage to the gastrointestinal mucosa [21,131]. However, there are very few studies in this area.

Insects are known to be one of the major sources of antimicrobial peptides (AMP). One of the insect defense mechanisms against pathogens and parasites results in the synthesis of AMPs or polypeptides produced by the fat body (equivalent to the mammalian liver) and certain blood cells. As shown in *Drosophila*, the response is generated via two separate pathways: the immune-deficiency (IMD) pathway initiated by Gram-negative bacteria and the Toll-receptor pathway stimulated by Gram-positive bacteria, yeasts, molds, and fungi [132]. Biologically active peptides have been found to exhibit antibacterial, antifungal, and antiviral properties. Insect proteins are precursors to such AMPs as defensins, cecropins, attacins, lebocins, and other proline-rich peptides, e.g., gloverins and moricins. Generally, insect antimicrobial peptides can be classified into four groups: α-helical peptides, cysteine-rich peptides, proline-rich peptides, and glycine-rich proteins [133]. Examples of antimicrobial insect peptides include termicin from termites, drosomycin from *Drosophila melanogaster*, heliomicin from the tobacco budworm (*Heliothis virescens*), and gallerimycin from greater wax moth larvae (*G. mellonella*) [132]. The amount of AMPs in insects varies significantly in different species and may have various types of activities, such as production of reactive oxygen species, inhibition of protein synthesis and permeabilization, and rupture or change of the electrochemical gradient of the membrane [133]. Most insect antimicrobial peptides have high activity against Gram-positive bacteria but lower activity against fungi, Gram-negative bacteria, and yeasts [132]. They usually have a similar profile: they are small (30–60 amino acid), strongly cationic (pI 8.9–10.7), heat stable (15 min in 100 °C) peptides with no resistance to drugs and no effect on eukaryotic cells [134]. Moreover, different interactions between antimicrobial peptides may occur, which are greater than additive antimicrobial effects, e.g., potentiation when one AMP facilitates or enhances the activity of others, or functional diversification, i.e., combinatorial activity [135].

Antioxidant peptides are the most popular group of insect peptides. The mechanism of the antioxidant action of peptides is not well understood, but some amino acids, such as H, P, W, and Y, have antioxidant activities, and antioxidant peptides commonly have these amino acids in their sequences [133]. As reported by Da Rocha et al. [136], hydrophobic and aromatic amino acids, e.g., histidine, methionine, tyrosine, lysine, and cysteine, enhance the potency of antioxidant peptides through proton-donation ability, electron-donation ability, and/or direct lipid radical scavenging ability. Moreover, Liu et al. [137] have found that low molecular weight peptides have more amino acids exposed to interact with free radicals, which improves their antioxidant effect. Hall et al. [138] has confirmed that the high scavenging capacities of insect hydrolysates can be an effect of the activity of smaller molecular weight peptides (di- or tripeptides) with better antioxidant potential. Nevertheless, Zielińska et al. [21] have shown high antioxidant activity of peptides composed of several amino acids (6–9 amino acids). Generally, the antioxidant activity of edible insect protein hydrolysates or single peptides was reported to be relatively high compared to that of other food protein hydrolysates [121].

Peptides from various species of edible insects, e.g., crickets (Amphiacusta annulipes, Gryllodes sigillatus), cockroach (Blaptica dubia, Gromphadorhina portentosa), locust (Locusta migratoria, Schistocerca gregaria), beetle (Zophobas morio, Tenebrio molitor), or butterfly larvae (Spodoptera littoralis, Bombyx mori) [21,131,133,139] were reported to have antioxidant activities. The antioxidant activities of peptides and hydrolysates were evaluated using free radical-scavenging activity (ABTS and DPPH methods), ion chelating activity, and reducing power assays. Alcalase™, thermolysin, or other proteases and simulated gastrointestinal digestion hydrolysates yielded antioxidant activity [121]. Furthermore, it has been shown that the heat treatment of insects before hydrolysis positively influences the antioxidant properties of peptides derived from their proteins [21,131]. To conclude, the bioactive potential of edible insect peptides appears to be similar or higher than that of other common food proteins. Therefore, these peptides with antioxidant, antimicrobial, anti-inflammatory, antidiabetic, and antihypertensive properties provide a wide range of applications for insect protein.

### 5.2. Peptides from Seafood By-Products

Proteins from fishery and seafood products are well recognized and have been confirmed to have high nutritive values, which can provide health benefits [140,141,142]. The demand for seafood is rising all over the world, driven by the increase in the population as well as awareness of the health benefits associated with seafood consumption [140]. Significant amounts of seafood are discarded annually during industrial scale processing operations. Additionally, processing of these products generates enormous amounts of by-products (50–80%) [140,143,144]. The discarded remnants, which seem to be inedible (e.g., heads, skin, viscera, scales, bones, etc.), contain as much as 60% protein on a dry weight basis [140,145,146], which are a rich source of constituents with bioactive effects [140,143,147]. Therefore, it is advisable to search for health-promoting ingredients in waste from the seafood and fishing industries [148,149].

It has been proved that seafood proteins exhibit various bioactivities including antioxidant [150,151,152,153,154,155,156], neuroprotective [157], antidiabetic [158], ACE inhibitory [159,160,161], DPP-IV inhibitory [162], immunomodulatory [163,164], antibacterial [165,166,167,168,169,170], cholecystokinin release regulating [171], antiproliferative [172,173], and anticancer activities [155,174]. ACE inhibitory and antioxidant peptides are two the most frequent types of peptides obtained from various seafood by-products. Two peptides, GASSGMPG and LAYA, have been purified from Pacific cod (*G. macrocephalus*) skin gelatin via pepsin hydrolysis [161]. Molecular docking has shown the potential of these peptides as ACE inhibitors with potential use in preparation of functional food targeted at lowering blood pressure and reducing the risk of CVDs [161]. The same result was reported for the IVDR, WYK, and VSAVI peptides obtained from olive flounder (*P. olivaceus*) surimi [175] and the LSGYGP peptide obtained from tilapia (*O. niloticus*) skin gelatin protein hydrolysate [176]. The LWHTH peptide obtained from the tunicate (*S. clava*) in in silico simulations was found to bind to the active site of ACE, making the ACE–LWHTH complex stable. Furthermore, LWHTH significantly reduced blood pressure in hypertensive rats [160]. The reported ACE inhibitory peptides identified from fish and seafood by-products have molecular weight ranging from 300 to 3000 Da, with the majority having from 2 to 13 amino acids in the sequence [177]. The activity of other enzymes was reported to be inhibited by seafood by-product peptides, e.g., peptides obtained from Atlantic salmon (*S. salar*) contributed to DPP-IV inhibition [159,162]. A bluefin leatherjacket (*N. septentrionalis*) by-product (head protein) was a source of three antioxidant peptides (WEGPK, GPP, GVPLT) [152]. Peptides showing antioxidant activity were also identified in *N. septentrionalis* skin (GSGGL, GPGGFI, FIGP) [153], skipjack tuna (*K. pelamis*) bones (GPDGR, GADIVA, GAPGPQMV, AGPK, and GAEGFIF) [150], tilapia (*O. niloticus*) skin (GIV, GAP*GF, GFA*GPA, SGNIGFP*GPK, GIPGPIGPP*GRP) [178], thornback ray (*R. clavata*) skin (GIPGAP) [154], and grass carp (*C. idella*) skin (PYSFK, GFGPEL, VGGRP) [157,179]. Seafood by-product-derived collagen peptides are specific protein fragments with health-promoting properties. They support the immune system and prevent cardiovascular and nervous diseases [180,181]. In food, seafood-derived collagen is used as a functional and nutritional ingredient for the development of health-enhancing foods.

Studies on seafood protein hydrolysates and peptides have reported effective anticancer activity [177,182]. A study of purified oyster peptides proved their anticancer activity against a colon cancer cell line (HT-29) [155]. Hydrolysates prepared from rainbow trout (*O. mykiss*) protein with the use of Flavourzyme and Alcalase enzymes showed their antioxidant and anticancer potentials [183]. Polypeptides extracted from oyster shells were found to inhibit tyrosinase activity [144].

The research focused on the characterization and isolation of antimicrobial peptides from fish processing by-products is less profuse than the investigations of antioxidant peptides. Antimicrobial peptides are chains of amino acids mostly with molecular weight below 10 kDa and containing less than 50 amino acids [184]. Most antimicrobial peptides from seafood have antibacterial activities against both Gram-negative and Gram-positive strains [167] due to their content of pardaxin, misgurin, cathelicidins, defensins, hepcidin, NK-lysin, piscidin, and trematocine with highly promising activities [168,169,170]. Potentially, these antimicrobial peptides are new candidates for the development of antibiotics in the pharmaceutical industry as well as antimicrobial agents for food production. These antimicrobial peptides can be used as antibacterial, antiviral, antifungal, immunomodulatory, and antitumor agents [167,184].

Given the diversity and complexity of seafood by-products, these results suggest that peptides extracted from seafood and their by-products can be used as ingredients for functional foods with great potential to improve consumer health. All described peptides are shown in Table 5.

### 5.3. Peptides from Seeds and Plants

The development of natural antioxidants from plant sources has become more important in the food and biological research arena. Studies conducted over the last five years have discovered various new peptides from different plant sources.

Plum (*P. domestica*) seeds are cheap sources of highly antioxidant and ACE inhibitory peptides [188]. Flaxseed (*L. usitatissimum*) protein and flaxseed-derived peptides from flaxseed meal have been documented to have physiological activity, e.g., angiotensin-converting enzyme (ACE) inhibition, antibacterial activity, antidiabetic effect, and antioxidant capacity [189]. Wild hazelnut (*C. heterophylla*) was a source of six peptides (ADGF, AGGF, AWDPE, DWDPK, ETTL, and SGAF) with excellent antioxidant activity [185]. A peptide (LAYLQYTDFETR) purified from pecan meal exhibited appreciable scavenging activities against the ABTS radical, DPPH radical, and hydroxyl radical (67.67%, 56.25%, and 47.42%, respectively) at 0.1 mg/mL [186]. A novel antioxidative peptide SMRKPPG was successfully purified and identified from peony (*P. suffruticos*) seed protein hydrolysate; the peptide exhibited effective antioxidative capacity in vitro [187]. The protein hydrolysate purified from amaranth (*Amaranthus*) seeds was shown to have antioxidant activity and anticancer potential against breast cancer cells [190]. Chia (*S. hispanica*) seeds are known for their high antioxidant capacity, which is related to the high content of phenolic compounds [191]. Moreover, antioxidant properties were evaluated in chia expeller peptides [192].

## 6. Conclusions

**Figure 3:**
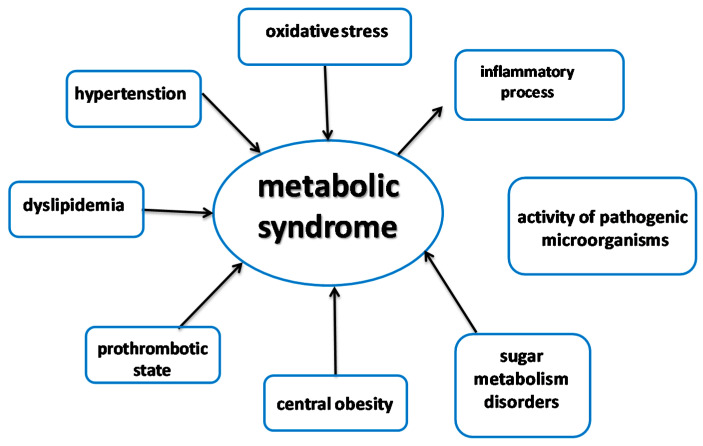
Risk factors of metabolic syndrome.

Biologically active peptides are the object of many studies, since nowadays, food is regarded not only as a dietary and nutrient source but also as a source of bioactive compounds that may provide health benefits and inhibit development of diseases. Bioactive peptides have different activities, with the main ACE inhibitor, antioxidant, antidiabetic, antiobesity, or antimicrobial effects (Figure 3). They can be obtained from several sources, also from new foodstuffs or residues from food production. Although much is known about the structure and activity of peptides, further studies on the relationship between their structure and activity must be continued. Furthermore, in addition to determination of their structure and activity in vitro, their therapeutic effect should be accurately determined, which will certainly be studied comprehensively in the future. The study results reported by researchers, nutritionists, and food manufacturers may improve the biodigestibility and bioavailability of food ingredients, such as peptides, and yield new functional foods supporting the pharmacotherapy of many civilization diseases.

## Figures and Tables

**Table 1:** Peptides with different activities.

Sequence of Peptide	Source of Peptide	Activity	IC50	Reference
WESLSRLLG	Ostrich egg white protein	ACE inhibitory	46.7 µg/mL	Asoodeh et al. [65]
		Antiradical against DPPH	15 µg/mL	
		Antiradical against ABTS	130 µg/mL	
		Anti-superoxide radical	150 µg/mL	
		Anti-hydroxyl radical	160 µg/mL	
GAA GFVG GIISHR ELLI KFPE	Spotless smoothhound muscle	Antiradical against ABTS	1.75 mg/mL 1.30 mg/mL, 0.34 mg/mL 0.32 mg/mL 0.46 mg/mL	Wang et al. [66]
SSEDIKE	Amaranth proteins	Anti-inflammatory activity	nd	Moronta et al. [67]
NMAINPSKENLCSTFCK	Casein proteins	ACE inhibitory	129.07 μM	Tu et al. [40]
NLEIILR TQMVDEEIMELFR	Mare whey protein	Dipeptidyl peptidase-IV inhibitory	86.34 μM 69.84 μM	Song et al. [68]
GGSK ELS	Red seaweed	α-amylase inhibitory	2.58 mM 2.62 mM	Admassu et al. [68]
KKFFRAWWAPRFLK	Synthetic peptides	Inhibition of the yeast *Zygosaccharomyces rouxii*	MIC (400 μg/mL)	Shwaiki et al. [69]
TTFHTSGY GYDTQAIVQ	Whey protein	ACE inhibitory	142 μM 1 mM	Villadóniga et al. [70]
YAP VIIF MAW	Cuttlefish muscle (*Sepia officinalis*)	ACE inhibitory	6.1 µM 8.7 µM 16.32 µM	Balti et al. [71]
ASPYAFGL	Mushrooms	ACE inhibitory	1.080 × 10^−7^ mol/L	Zhang et al. [72]
AREGEM	Synthetic peptide	Antioxidant	nd	Cao et al. [73]
LAHMIVAGA VAHPVF	Quinoa yoghurt beverages	α-glucosidase inhibitory	127 mg/mL 10.39 mg/mL	Ujiroghene et al. [74]
HGSEPFGPR RGDPFPWPWYSH RPRYPWRYT	Amaranth proteins	LOX inhibitory	11.5 µM >50 µM 17.3 µM	Montoya-Rodríguez et al. [75]

nd—not determined.

**Table 2:** Peptide sequences with antioxidant activity.

Sequences of Peptide	Antioxidant Methods	Source of Peptide	Antioxidant Activity Expressed as: IC _50_; % or Trolox Equivalent	Reference
LDDPVFIH VAAGRTDAGVH	DPPH radical scavenging ABTS radical scavenging reducing power	fermented anchovy fish (Budu) extract	0.84 mg/mL 1.45 mg/mL 0.617 mg/mL 0.795 mg/mL 0.702 0.422	Najafian and Babji [72]
VVEVYLPR, VEVYLPR, VYLPR	ORAC	egg-white	36.09 µM 41.05 µM 44.37 µM	Zhang et al. [76]
IREADIDGDGQVN, PEILPDGDHD, ASDEQDSVRL, APLEEPSSPH	DPPH radical scavenging DPPH radical scavenging DPPH radical scavenging Fe^2+^ chelating ability	crucian carp	1.78 mM 1.18 mM 1.45 mM 0.09 mM	Zhang et al. [66]
TSSSLNMAVRGGLTR, STTVGLGISMRSASVR	DPPH radical scavenging	finger millet	80.55% 75.1%	Agrawal et al. [79]
SYPTECRMR	DPPH radical scavenging ABTS radical scavenging	sesame	0.105 mg/mL 0.004 mg/mL	Lu et al. (2019) [71]
QMDDQ	DPPH radical scavenging, hydroxyl radical-scavenging activities	shrimp	0.5 mg/mL 1.0 mg/mL	Wu et al. [74]
EVGK, RCLQ	Fe^2+^ chelating ability reducing power, ABTS radical scavenging DPPH radical scavenging	duck plasma	16.35% 0.62, 274.83 mM 95.12%	Yang et al. [73]
LAGNPHQQQQN and HNLDTQTESDV	hydroxyl radical scavenging or ROS reduction	walnut meal	-	Sheng et al. [66]
SF and QY	protective effects on 385 H_2_O_2_-induced Chang liver cells.	*M. oleifera* seed	-	Liang et al. [70]
LY, RALP and GHS	inhibited the production of ROS and lipid peroxide	rapeseed	-	He et al. [84]
WDHHAPQLR	model of Caco-2 cell monolayers and oxidative stress in HUVECs	rapeseed	-	Xu et al. [83]
NTVPAKSCQAQPTTM, EDELQDKIHPF, QGPIVLNPWDQVKR, APSFSDIPNPIGSENSE	model of Caco-2 cell	fermented milk	-	Tonolo et al. [85]
AGPSIVH, FLLPH, LLCVAV	DPPH radical scavenging ABTS radical scavenging reducing power	duck breast	56.41% 0.6393 mmol TE/g 0.0651	Li et al. [80]
LLSGTQNQPSFLSGF, NSLTLPILRYL, TLEPNSVFLPVLLH	ORAC	lentil storage proteins	0.013 μmol TE/μmol 1.432 μmol TE/μmol 0.139 μmol TE/μmol	García-Mora et al. [75]
AYL AYI	ORAC	Jiuzao	1.35 μmol TE/μmol 1.37 μmol TE/μmol	Jiang et al. [70]
Peptide fractions *<* 1 kDa	DPPH, ABTS, hydroxyl radical-scavenging activities	brown rice	0.19 mM TE, 2.28 mM TE, 24.64 mM TE,	Selamassakul et al. [63]

**Table 3:** Peptide sequence with antimicrobial activity.

Sequence of Peptide (Name)	Source of Peptide	Antimicrobial Activity	Reference
RYRRKKKMKKALQYIKLLKE (peptide 35,409)	synthetic peptide, analog from peptide 20,628 (321RYRRKKKMKKKLQYIKLLKE340)	inhibit growth of *E. coli*, *S. aureus, P.aeruginosa*	Barreto-Santamaría et al. [104]
ASHLGHHALDHLLK (H2)	*Holothuria tubulosa*	inhibit growth of *L. monocytogenes*	Cusimano et al. [105]
MRGSHHHHHHGSSGENLYFQSL (Tag)	synthetic peptide	inhibit growth of *L. monocytogenes*	Cusimano et al. [105]
GIWKKWIKKVVNVLKNLF-NH_2_ (KU2)	hybride peptides (KABT-AMP/Uperin 3.6)	inhibit growth of *C. albicans*	Lum et al. [110]
GIWKKWIKKWLNVLKNLF-NH_2_ (KU3)	hybride peptides (KABT-AMP/Uperin 3.6)	inhibit growth of *C. albicans*	Lum et al. [110]
KTCENLADTYKGPPPFFTTG (phaseococcin)	*Phaseolus coccineus*	inhibit HIV reverse transcriptase activity	Patrick et al. [111]
KTCENLADTY (sesquins)	*Vigna sesquipedalis*	inhibit HIV reverse transcriptase activity	Wong and Ng [112]

**Table 4:** Peptide sequence with ACE inhibitor active.

Sequence of Peptide	Source of Peptide	Activity	Reference
KHV	*Bombyx mori*	ACE inhibitory	Jia et al. [126]
ASL	*Bombyx mori*	ACE inhibitory	Wu et al. [127]
GNPWM	*Bombyx mori*	ACE inhibitory	Tao et al. [124]

**Table 5:** Bioactive peptides obtained from seafood by-products.

Sequence of Peptide	Source of Peptide	Activity	Reference
Seafood by-products			
GASSGMPG LAYA	*Pacific cod (G. macrocephalus*)	ACE inhibitory	Ngo et al. [161]
IVDR WYK VSAVI	olive flounder (*P. olivaceus*) surimi	ACE inhibitory	Oh et al. [175]
LSGYGP	tilapia (*O. niloticus*) skin	ACE inhibitory	Chen et al. [176]
LWHTH	tunicate (*S. clava*)	ACE inhibitory	Kang et al. [160]
YP	Atlantic salmon *(S. salar)*	DPP-IV inhibitory	Neves et al. [159]
WEGPK GPP GVPLT	Bluefin leatherjacket (*N. septentrionalis*) head	antioxidant	Chi et al. [152]
GSGGL GPGGFI FIGP	*N. septentrionalis* skin	antioxidant	Chi et al. [153]
GPDGR GADIVA GAPGPQMV AGPK GAEGFIF	skipjack tuna (*K. pelamis*) bones	antioxidant	Yang et al. [150]
GIV GAP*GF GFA*GPA SGNIGFP*GPK GIPGPIGPP*GRP	tilapia (*O. niloticus*) skin	antioxidant	Thuanthong et al. [178]
GIPGAP	thornback ray (*R. clavata*) skin	antioxidant	Lassoued et al. [154]
PYSFK GFGPEL VGGRP	grass carp (*C. idella*) skin	antioxidant	Cai et al. [157] Cai et al. [179]
Plants and seeds			
ADGF AGGF AWDPE DWDPK ETTL SGAF	Wild hazelnut (*C. heterophylla*)	antioxidant	Liu et al. [185]
LAYLQYTDFETR	pecan meal	antioxidant	Hu et al. [186]
SMRKPPG	peony (*P. suffruticos*) seed	antioxidant	Zhang et al. [187]
